# Comprehensive single-cell transcriptome analysis reveals heterogeneity in endometrioid adenocarcinoma tissues

**DOI:** 10.1038/s41598-017-14676-3

**Published:** 2017-10-27

**Authors:** Shinichi Hashimoto, Yuta Tabuchi, Hideaki Yurino, Yoshihiko Hirohashi, Shungo Deshimaru, Takuya Asano, Tasuku Mariya, Kenshiro Oshima, Yuzuru Takamura, Yoshiaki Ukita, Akio Ametani, Naoto Kondo, Norikazu Monma, Tadayuki Takeda, Sadahiko Misu, Toshitugu Okayama, Kazuho Ikeo, Tsuyoshi Saito, Shuich Kaneko, Yutaka Suzuki, Masahira Hattori, Kouji Matsushima, Toshihiko Torigoe

**Affiliations:** 1 Department of Integrative Medicine for Longevity, Graduate School of Medical Sciences, Kanazawa University, Ishikawa, 920-8641 Japan; 2 CREST, Japan Science and Technology Agency, Tokyo, 102-0076 Japan; 3 Department of Pathology, School of Medicine, Sapporo Medical University, Hokkaido, 060-0061 Japan; 4 Department of Molecular Preventive Medicine, Graduate School of Medicine, The University of Tokyo, Tokyo, 113-0033 Japan; 5 Department of Computational Biology, Graduate School of Frontier Sciences, The University of Tokyo, Chiba, 277-8561 Japan; 6 Department of Bioscience and Biotechnology, Japan Advanced Institute of Science and Technology, Ishikawa, 923-1292 Japan; 7 Faculty of Engineering, Graduate Faculty of Interdisciplinary Research, University of Yamanashi, Yamanashi, 400-8511 Japan; 8 RIKEN Center for Life Science Technologies, Kanagawa, 230-0045 Japan; 9 Laboratory of DNA Data Analysis, National Institute of Genetics, Shizuoka, 411-8540 Japan; 10 Department of Obsterics and Gynecology, School of Medicine, Sapporo Medical University, Hokkaido, 060-0061 Japan; 11 Department of Disease Control and Homeostasis, Faculty of Medicine, Kanazawa University, Ishikawa, 920-8641 Japan; 12 Graduate School of Advanced Science and Engineering, Waseda University, Tokyo, 169-8555 Japan

## Abstract

Single cell transcriptome analysis of a cancer tissue can provide objective assessment of subtype population or the activation of each of various microenvironment component cells. In this study, we applied our newly developed technique of single cell analysis to the myometrial infiltration side (M-side) and the endometrial side (E-side) of a human endometrioid adenocarcinoma with squamous differentiation tissues. We also analyzed spherogenic cultures derived from the same tissue to identify putative regulators of stemness *in vivo*. Cancer cells in the E-side were highly malignant compared with those in the M-side. Many cells on the E-side were positive for spheroid-specific tumorigenesis-related markers including *SOX2*. In addition, there were higher numbers of epithelial-to-mesenchymal transition (EMT) cells in the E-side compared with the M-side. This study identified a site containing cells with high malignant potential such as EMT and cancer stem-like cells in cancer tissues. Finally, we demonstrate that established endometrioid adenocarcinoma subtype classifiers were variably expressed across individual cells within a tumor. Thus, such intratumoral heterogeneity may be related to prognostic implications.

## Introduction

As tissues are rarely homogeneous, gene expression profiles from a single tissue sample are the sum of the profiles for each type of constituent cell. The relative contribution of each cell type is unknown and the expression profile of each is also uncertain. One possible means of surmounting these problems is the use of single-cell gene-expression profiling to enable the identification and characterization of different cell types and subtypes. However, scalable approaches are needed to characterize complex tissues with a range of cell types and states under diverse conditions. To overcome this problem, single-cell methods have been developed for microarrays and RNA-seq for gene expression analyses^1,2^. Although commercially available microfluidics technologies are commonly used to analyze the mRNA expression in hundreds of individual cells^3^, these techniques require expensive equipment and involve considerable time and effort for the treatment of cells. Recently, several methods for single cell gene expression analysis^4–8^ that enable measurement of considerably larger numbers of single cells have been developed. We have also developed a novel strategy called Next generation 1 cell sequencing (Nx1-seq for short) to enable the single cell transcriptome analysis of thousands of single cells. Nx1-seq makes use of barcoded beads that can easily be amended to include a particular sequence. This new approach has high sensitivity and good reproducibility, and does not require expensive equipment.

The tumor microenvironment consists of many different cell types that have a range of biological roles. Navin *et al*.^9^ reported that analysis of 100 single cells from a polygenomic tumor revealed three distinct clonal subpopulations that probably represented sequential clonal expansions. Cancer development is influenced by differential microenvironmental conditions such as hypoxia, immune cell infiltration and stromal fibrosis. Single-cell analysis in cancer can provide an objective assessment of the overall abundance and activation of various microenvironment components, including cancer-associated fibroblasts, vessels and immune cells.

In this study, we used Nx1-seq to characterize the complex cellular heterogeneity in a human endometrioid adenocarcinoma tissue. Endometrial cancer is the most common global the gynecologic malignancy^10,11^. Approximately 80% of cases are estrogen-dependent well-to-moderately differentiated endometrioid endometrial carcinomas (EEC) that are usually confined to the uterine corpus at diagnosis of stage I and, thus, most are surgically curable. In contrast, estrogen-independent EECs are poorly differentiated tumors that frequently invade the myometrium, extend beyond the uterus at the time of hysterectomy, and are associated with a poor outcome. Despite the high and increasing incidence of endometrial cancer, our current models for the prediction of prognosis and treatment responses are suboptimal. Novel molecular biomarkers are needed to assist clinical decisions. This study used our Nx1-seq analysis method to identify each cell type and status in a cancerous tissue and to develop candidate biomarker molecules.

## Results

### Sensitivity and reproducibility and of Nx1-seq

**Figure 1:**
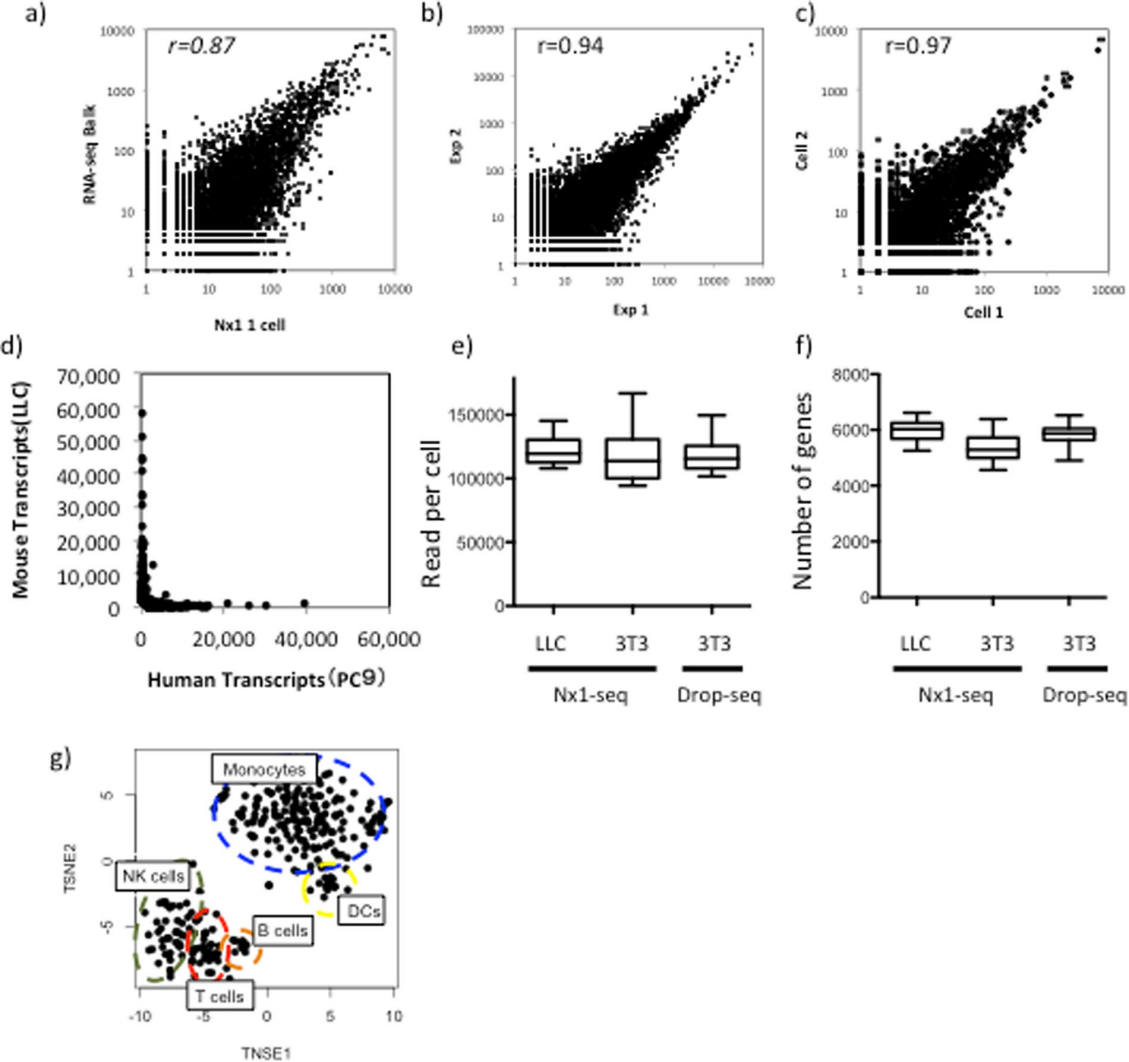
Sensitivity and reproducibility and of Nx1-seq. (**a**) Correlation between gene expression analysis in Nx1-seq and bulk serial analysis of gene expression methods with the PC9 cell line. The correlation was performed using log transformed data. (**b**) Comparison of Nx1-seq gene expression measurements (average of 50 single PC9 cell data) between two independent experiments. (**c**) Comparison of Nx1-seq gene expression measurements between two independent PC9 cells. (**d**) Nx1-seq analysis of mixtures of human and mouse cells. Mixtures of human (PC9) and mouse (LLC) cells were analyzed by Nx1-seq. The scatter plot shows the number of human and mouse transcripts associated with each read/cell. (**e**)Number reads and (**f**) genes detected in single-cell libraries generated by Nx1-seq or Drop-seq (ref.^8^). Using Nx1-seq (Drop-seq), an average depth of 119,833 per LLC cell and 124,290 (118,691) reads 3T3 cell or 5,919, 5,363 (5,847) genes were detected among mouse LLC and NIH3T3 cells (n = 50 for Nx1-seq; n = 50 for Drop-seq). (**g**) Clustering of 300 human peripheral blood mononuclear cells (PBMC) analyzed by Nx1-seq.

We have developed a method for comprehensive single-cell transcriptome analysis, which can obtain digital gene expression data from thousands of individual cells (Supplementary Fig. 1a, b). To confirm the ability of Nx1-Seq to identify individual cells such as the PC9 cells, a lung cancer cell line, we compared the results with those from the RNA-seq analysis of a bulk cell population (hundreds of thousands of cells) (Fig. 1a and Supplementary Table 1). We also performed Nx1-seq with the PC9 cell line to ensure the reproducibility of technical replicates. The top 100 libraries from PC9 cells were compared to the bulk cell library, and Pearson correlation coefficients of approximately 0.94 were obtained (Fig. 1b). The pooling of Nx1-seq data from single cells of two homogeneous cell populations provided rich and highly reproducible transcriptional profiles. In addition, gene expression patterns among libraries with large sequencing reads were similar (r = 0.97), as shown in the mean of the scatter plots (Fig. 1c). We tested the ability of Nx1-seq to identify individual cells among two cell types using a ~1:1 mixture of PC9 (a human lung carcinoma cell line) and LLC (a mouse lung cancer cell line) cells. The cells were loaded onto a microwell and Nx1-seq was performed using a Miseq sequencer. We found that human and mouse transcripts were associated with each cell barcode (Fig. 1d). Next, Nx1-seq data was compared with an other massively parallel bead-based scRNA-seq method, Drop-seq using mouse cell lines. The gene detection levels per reads of Nx1-seq for each cell lines were similar to those of Drop-seq (Fig. 1e,f). Moreover, we tested the method on human peripheral blood mononuclear cells. As shown in Fig. 1g and Supplementary Fig. 2, the obtained libraries could be clustered into monocytes, T cells, NK cells and B cells by a three dimensional analysis using t-Distributed Stochastic Neighbor Embedding (tSNE). These data suggest that Nx1-seq can be used to identify genes expressed in each cell population.

### Heterogeneity of tumor tissues

**Figure 2:**
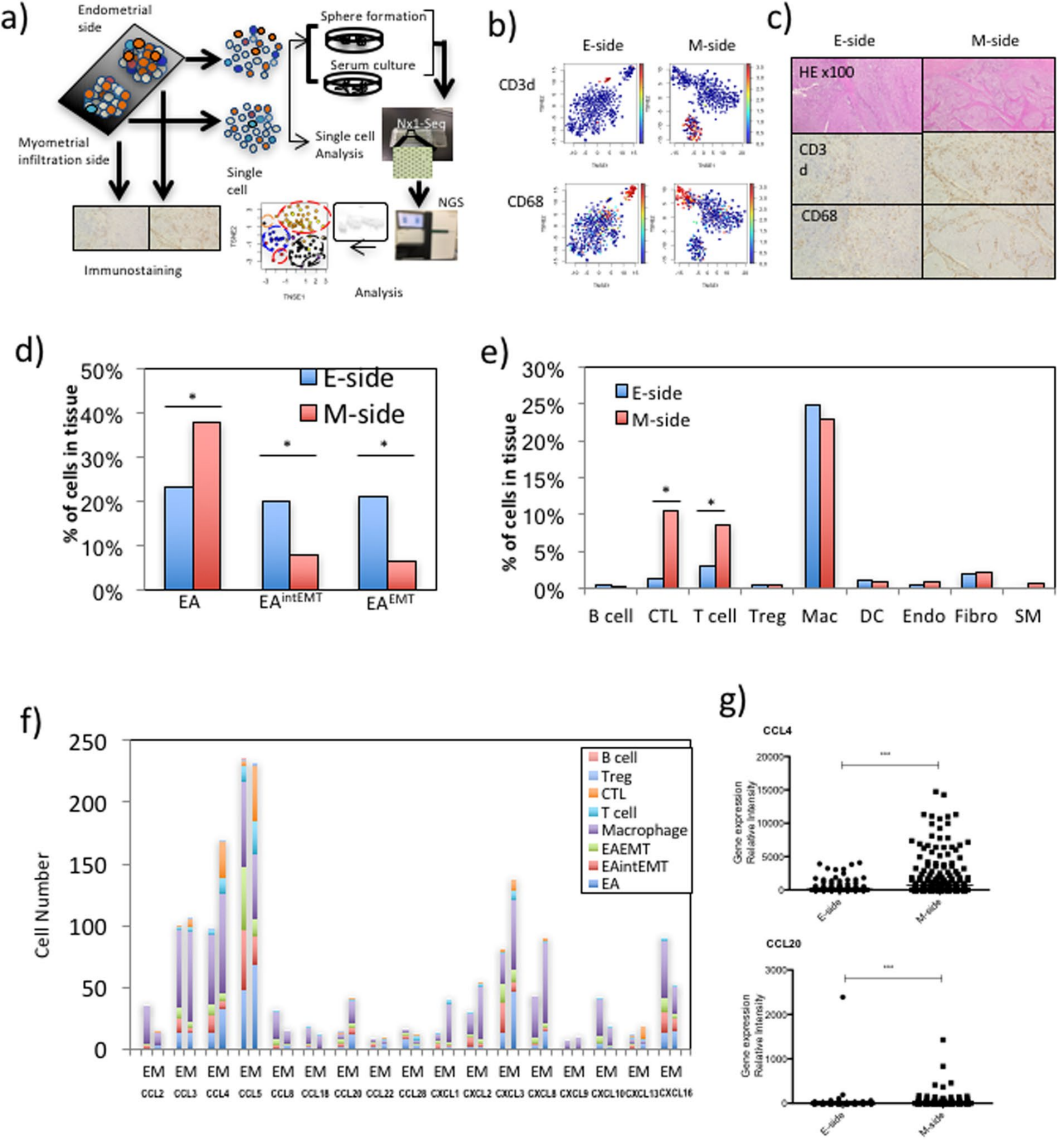
Heterogeneity of tumor tissues. (**a**) The workflow shows the rapid dissociation of cells from two sites of a primary tumor to generate single cells and sphere cells. The obtained cells were then analyzed by Nx1-seq and by histopathological analysis. (**b**) Clustering analysis. t-SNE map representation of transcriptome similarities between individual cells. The upper two panels represent T cells (red) from the E-side and the M-side, while the lower two panels represent macrophages (red) from each side. (**c**) Immunohistostaining of endometrial adenocarcinoma at the myoinvasive state. The endometrial side and myoinvasive tumor side were stained using specific antibodies. (**d**) Heterogeneity of cancer cells. EA, EA^intEMT^ and EA^EMT^ cells were defined as described in the Materials and Methods. *p < 0.05 for the E-side versus M-side by the Mann–Whitney *U*-test. (**e**) Cells infiltrating into tumor tissues. These cells were defined as described in the Materials and Methods. *p < 0.05 for the E-side versus M-side by the the Mann–Whitney *U*-test. (**f**) Chemokine production in cancer cells and infiltrating immune cells in each side. (**g**) Comparison of CCL4 and CCL20 expressing cells between the E-side and the M-side. These data were calculated using cells extracted from defined cell data shown in Supplementary Table 2. ***p < 0.001 for the E-side versus M-side by the Mann–Whitney *U*-test.

Endometrioid adenocarcinoma (EA) of the endometrium is the most common type of endometrial carcinoma^10^. In this study, we obtained single cell transcriptomes from an EA with squamous differentiation. Figure 2a shows the workflow of Nx1-seq. To estimate the endometrial tumor cell diversity, a single cell analysis was performed using Nx1-seq. We also analyzed the differences between cancer tissue cell populations from two sites, namely the endometrial side (E-side) and the myometrial infiltration side (M-side). Myometrial invasion is an independent prognostic marker of endometrioid carcinomas and correlates with the risk of metastasis to pelvic and/or para-aortic lymph nodes^10,12^.

One thousand cells from the E-side and from the M-side were analyzed and yielded more than 400 genes per cell and more than 20,000 reads (Supplementary Table 2). We performed tSNE for cells in the E-side and M-side, and showed three major clusters based on cell types. The cell populations inferred from this analysis were readily matched to the tumor cells and infiltrating lymphocytes, including macrophages and T cell classes, based on the specific expression of known markers such as *CD3D* and *CD68* (Fig. 2b). We defined each cell type using several cell-specific genes as shown in Supplementary Table 3. When we compared the cellular ratio in the tissues of both sides, T cell infiltration in the M-side was higher than in the E-side (Fig. 2b). Next, to determine whether the gene expression pattern from single cells was consistent with the locations of individual tissue cells in the cancer tissue, EA tissue sections were stained with antibodies specific for immune cell markers such as CD4, CD8, TIA1, FOXP3 and CD20, cell proliferation marker such as BIRC5 and MKI67, stromal cell marker such as VIM and ACTA2 (Fig. 2c and Supplementary Fig. 3). These data showed that the Nx1-seq data of infiltrating T cells was consistent with the pathological data. Moreover, we estimated the population of the infiltrating T cells between the M-side and the E-side in EA from eight other endometrioid adenocarcinoma patients (Supplementary Fig. 4). In agreement with the previous experiment, the data showed that T cell infiltration in the M-side was higher than in the E-side. The relative abundance of the major cell classes in our data agreed with the pathological data, indicating that Nx1-seq provided an accurate assessment of the cell population in the tumor environment.

### Heterogeneity of cancer cells

We next applied the Nx1-seq method to the characterization of cancer cells. It is well known that cancer cell populations include cancer stem cells, differentiated cells in the mesenchyme transitioning from epithelial cells, and cells affected by therapies. Therefore, we sought to determine whether our method could differentiate these cell populations using a range of biomarkers despite the accumulation of gene mutations in endometrial cancer. We used estrogen receptor (ER) and progesterone receptor (PR) as prognostic biomarkers as these have been validated for endometrial cancer^11^. Loss of ER and PR is linked to aggressive tumors, specifically to the endometrioid subtype. In addition, *STMN1* and *HER2* overexpression identifies high-risk patients and lymph node metastasis in endometrial cancer^11^. In agreement with the pathological assessment, few cells on either side were found to express ER or PR. In contrast, *STMN1* positive cells were more abundant in the E-side (Supplementary Fig. 3).

**Figure 3:**
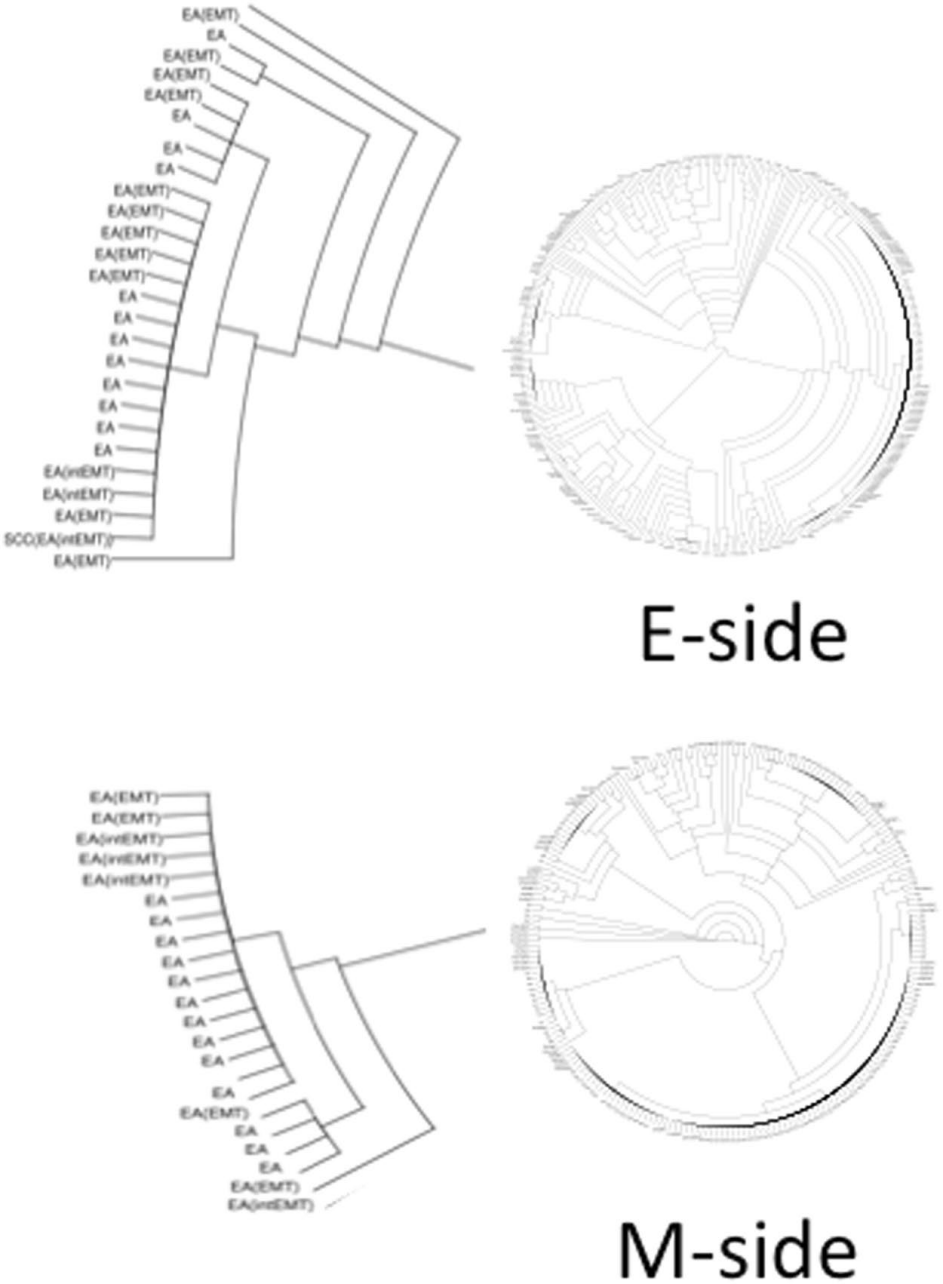
Clustering of cancer cells. We performed an unsupervised cluster analysis using the Nx1-seq data to determine to what degree the two sides of the cancer tissue could be distinguished for EA, EMT[intEMT] and EA[EMT] types. Notably, there was not complete separation of these three cancer types, indicating that each single cell became a single EA^EMT^ during the growth of cancer. Enlarged view shows one example.

Myometrial invasion in endometrioid carcinomas is thought to be correlated with the risk of metastasis and is related to epithelial-to-mesenchymal transition (EMT)^13–15^. We therefore used Nx1-seq to examine EMT in the E-side and M-side. We screened for cancer cells expressing at least one EMT marker, such as *ACTA2*, *EPCAM*, *CD44*, *THY1*, *VIM*, *FN1*, *ZEB1* or *CDH1* (Fig. 2d). Our results showed that the cancer cells could be separated into three groups as follows: cells with only epithelial markers (EA); cells with only mesenchymal markers (EA^EMT^); and cells with both epithelial markers and mesenchymal markers (EA^intEMT^). To characterize the cancer cells further, we used an unsupervised cluster analysis (Fig. 3). Interestingly, each cluster of cancer cells inferred from this analysis contained all three types of cells, namely EA, EA^intEMT^, and EA^EMT^ cells (Fig. 2d). These data suggested that EMT-like cells in the classified groups might be derived from a single cell.

The relative frequencies of different EMT-like cells in the E-side and M-side were estimated. The analysis indicated many EA type cells in the M-side. In contrast, the EA^intEMT^ and EA^EMT^ cell types contributed a higher proportion of EMT-like cells in the E-side compared with the M-side (Fig. 2d). Over all, the results indicated that the cancer cell populations in the E-side and M-side were different. In addition, we examined expression of specific genes in EA^EMT^ type cells. Genes that highly expressed in EA^EMT^ compared with EA type cells were *TCEAL4*, *BAG1*, *VAT1*, and *GPI* which are known to be gynecological tumor markers (Supplementary Table 4).

### Heterogeneity of infiltrating immune cells

**Figure 4:**
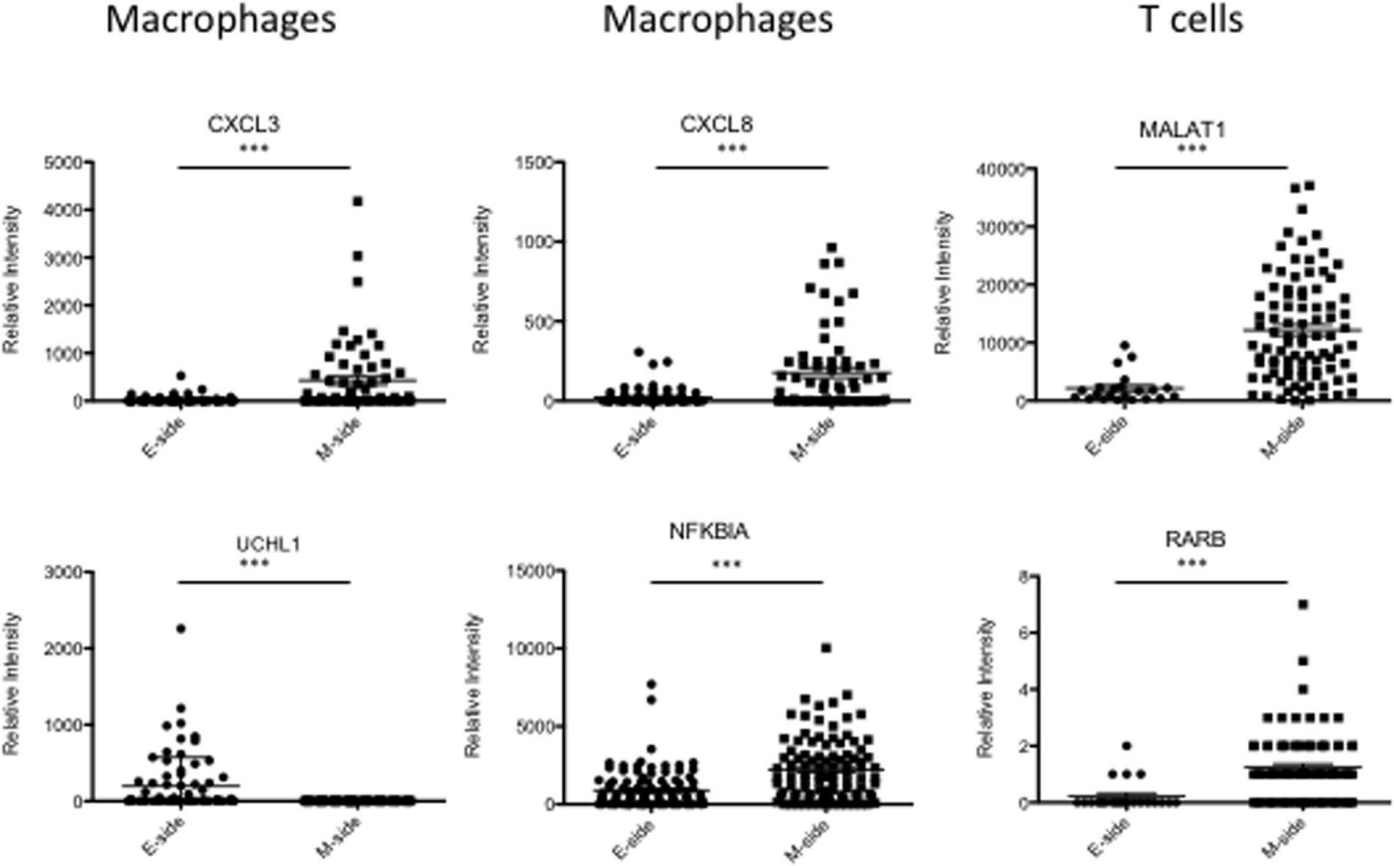
Differentially expressed genes in the infiltrating immune cells in the E-side and M-side. Comparison of each gene-expressing cell between the E-side and M-side. These data were calculated using cells extracted from defined cell data shown in Supplementary Table 2. ***p < 0.001 for the E-side versus M-side by the Mann–Whitney *U*-test.

Recently, attention has turned to the various types of non-neoplastic cells present in tumors, such as stromal cells and infiltrating leukocytes, which can interact with tumor cells and have a major influence on long-term outcome in patients^16,17^. In addition to CD8+ cytotoxic T cells, macrophage subsets are also considered a key component of effective antitumor immunity^18^. We therefore sought to characterize leukocyte infiltration of the endometrial cancer microenvironment using Nx1-seq to analyze gene expression in macrophages and T cells in the E- and M-sides (Fig. 2e). The population of T cells and cytotoxic T lymphocytes (CTLs) was larger in the M-side than the E-side. Although the ratios of tumor-infiltrating macrophages did not differ between the two sides there were some qualitative differences in macrophages. A population of macrophages expressing *CXCL3* and *CXCL8*, chemoattractants in inflammation, and *NFKBIA*, was present in greater numbers in the M-side (Fig. 4). In contrast, macrophages with *UCHL1*, which promotes cancer progression^19^, were more common in the E-side. The proportions of macrophages expressing the inflammatory factors *CCL5*, *IL10* and *IL6* did not differ between the two sides. The analysis of gene expression levels in T cells showed that *MALAT1*- and *RARB*-expressing T cells were more frequent in the M-side.

### Cells expressing chemokines in cancer tissues

The infiltration of macrophages and T cells into cancer tissues is regulated by several chemokines. We therefore investigated chemokine expression in tumor tissues by Nx1-seq. Figure 2f shows the number of cells expressing chemokines in the E- and M-sides. The number of cells expressing CCL4, CCL20, CXCL1, and CXCL2 was greater in the M-side than the E-side. Cells expressing these chemokines consisted of cancer cells including EA, EA^intEMT^ and EA^EMT^ cells, as well as macrophages and T cells. In addition, it is well known that CCL3, CCL4 and CCL20 are able to recruit T cells containing CTLs to the tumor site. Therefore, these chemokines might contribute to tissue invasion by T cells. Next, we examined the expression of CCL4 and CCL20 by T cells in the E- and M-sides (Fig. 2g). Our results indicated that chemokines were expressed by T cells and that their expression levels were different in the two sides.

### Characterization of spheroid cells established from endometrial adenocarcinoma tissues

**Figure 5:**
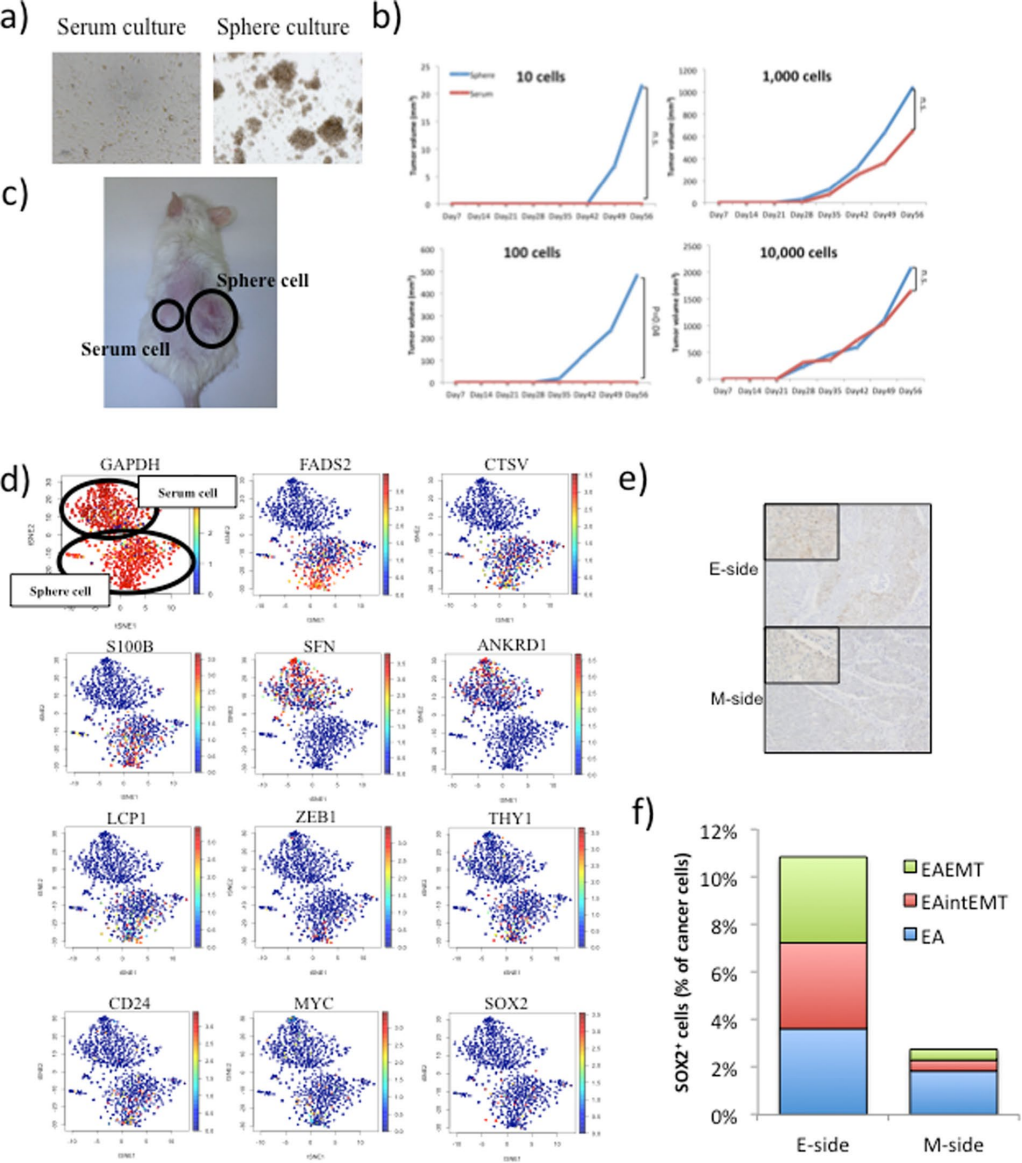
Characterization of sphere cells derived from the primary tumor and the patterns of gene expression. (**a**) Sph cells (sphere culture) and Ser cells (serum culture) were cultured in suspension for 1 week and then transferred to attachment conditions. (**b**) Tumor growth in mice injected with Sph cells and Ser cells. Data are shown as the means ± SD. All statistical analyses for data in this figure were performed using a paired Student’s *t*-test. (**c**) Tumorigenicity of Sph cells at the subcutaneous injection site. Sph cells and Ser cells were injected into NOD/SCID mice at 1 × 10^2^ cells/mouse. (**d**) t-SNE map representation of transcriptome similarities between individual Sph cells and Ser cells. Red dots show cells positive for the indicated gene. (**e**) Immunohistostaining for SOX2 positive cells in the E-side and the M-side. Original magnification 100x for the insert in the upper left corner and 40x for the remaining area. (**f**) Percentage of SOX2 positive cells among three types of cancer cells in the E-side and the M-side.

It has been reported that cancer stem/initiating cells are present in endometrial cancer^20,21^. Here, we used Nx1-seq analysis to determine whether this method could also detect cancer stem/initiating cells in endometrial cancer. To characterize cancer stem/initiating cells from endometrial adenocarcinoma cells, we used a sphere-forming assay to enrich potential stem cells from the endometrial tissue and then performed Nx1-seq analysis (Fig. 5a). In addition, we also analyzed sphere-derived cells growing in serum adherent cultures (Ser cells), because the use of serum promotes cell differentiation. Sphere-derived cells growing in serum-free nonadherent cultures (Sph cells) were used to examine whether they influenced *in vivo* tumor formation when transplanted into NSG mice, compared with Ser cells. NSG mice were injected with 1–10 × 10^5^ Sph and Ser cells. Subcutaneous tumor formation was induced by the injection of 10 Sph cells as shown by macroscopic examination of a tumor derived from Sph cells (Fig. 5b,c). In contrast, at least 1 × 10^2^ Ser cells were required to give rise to a tumor. These results support the hypothesis that Sph cells have a high cancer-initiating ability.

When a sample of 500 cells from Sph and Ser were analyzed by Nx1-seq, both cell types were highly heterogeneous as previously reported (Fig. 5d)^22^. The relative number of cells positive for each of the genes screened was then compared between Sph and Ser cells (Supplementary Table 5). In Sph cells, the gene expressed in the largest proportion of cells was *FADS2*, followed by *CTSV*, *S100B*, and *SCG2* (Fig. 5d). In contrast, the most frequently expressed gene in Ser cells was *SFN*, followed by *ANKRD1*, *KRTAP2*-3, and *CA2*. To identify gene-signature-based differences between Sph and Ser cell populations, we performed a gene set enrichment analysis (GSEA). Our results showed that gene sets involving “Nucleobasenucleosidenucleotide and nucleic acid metabolic process”, “biopolymer metabolic process” and “negative regulation of cellular process” were upregulated in Sph/Ser populations. Genes such as *MLF1*, *PITX2*, *GADD45G*, *CREB5* and *CDK2* are related to stem cell regulation. Moreover, *EMT* (*VIM*, *ZEB1* and *ACTA2*) and stem cell markers (*SOX2*, *POU5F1*, *NANOG*, *BMI1*, *THY1*, *CD24*, *CD44*, *PROM1* and *REXO1*) were also observed in Sph and Ser cells (Supplementary Table 5). *SOX2*, *THY1*, *CD24* and *ZEB1* genes were expressed at a higher level in Sph cells compared with Ser cells (Fig. 5d).

### Cancer tissues and spheroid cells

The distribution of cancer stem-like cells expressing Sph-specific genes was analyzed in the two sides of endometrial cancer tissues. A larger proportion of cells showing high levels of gene expression were found in the E-side than the M-side (Table 1); by contrast, higher levels of expression occurred more frequently in Ser cells were found in the M-side. Cells positive for the stem cell markers THY1, MYC and SOX2 occurred at a higher rate in the E-side. Figure 5e shows immunohistostaining for SOX2 positive cells in the E-side and the M-side; the results of this immunohistochemical analysis for SOX2 positive cells were consistent with the pathological data.

**Table 1:** Distribution of cells with spheroid specific genes in the E- and M-sides of endometrial adenocarcinoma tissue.

	Highly expressed genes in spheroid.				Fold (E/M)	Highly expressed genes in cells in serum culture					Fold (E/M)
	E-side	Ratio	M-side	Ratio			E-side	Ratio	M-side	Ratio	
	Number of cells		Number of cells				Number of cells		Number of cells		
FADS2	147	48%	55	31%	1.6	SFN	5	2%	18	10%	6.1
CTSV	41	13%	9	5%	2.7	ANKRD1	15	5%	37	21%	4.2
S100B	61	20%	3	2%	12.0	KRTAP2-3	1	0%	1	1%	1.7
SCG2	2	1%	0	0%	1.0	CA2	0	0%	0	0%	0.0
BAMBI	58	19%	28	16%	1.2	CPA4	4	1%	1	1%	0.4
DUSP6	64	21%	23	13%	1.6	EPHB3	1	0%	2	1%	3.4
LCP1	13	4%	3	2%	2.6	PLEK2	8	3%	2	1%	0.4
RBBP7	168	55%	63	35%	1.6	IGFBP3	2	1%	1	1%	0.8
QPCT	27	9%	22	12%	0.7	DMKN	62	20%	43	24%	1.2
SOX2	52	17%	10	6%	3.1	CAPG	51	17%	34	19%	1.1
THY1	44	14%	3	2%	8.7	TAGLN	11	4%	2	1%	0.3
ZEB1	4	1%	2	1%	1.2	FXYD5	21	7%	6	3%	0.5
BMI1	17	6%	6	3%	1.7	KRT19	76	25%	79	44%	1.8
CD24	141	46%	78	43%	1.1	ID4	73	24%	51	28%	1.2
NANOG	8	3%	11	6%	0.4	ARL4C	23	8%	23	13%	1.7
MYC	118	39%	39	22%	1.8	PFKP	19	6%	15	8%	1.3
PROM1	5	2%	14	8%	0.2	MT1L	18	6%	6	3%	0.6
RAB33A	22	7%	3	2%	4.3	ALDH1A1	24	8%	14	8%	1.0
GNG2	19	6%	1	1%	11.2	VPS13D	4	1%	4	2%	1.7
ACSL3	31	10%	14	8%	1.3	ID1	2	1%	0	0%	0.0
HMGCS1	38	12%	17	9%	1.3						
FXYD6	32	10%	4	2%	4.7						
MLF1	19	6%	2	1%	5.6						
ZRSR2	35	11%	10	6%	2.1						
PTH1R	1	0%	0	0%	0.0						
S100A1	8	3%	5	3%	0.9						
MVK	20	7%	5	3%	2.4						
TMIE	0	0%	0	0%	0.0						
SERPINF1	45	15%	16	9%	1.7						
THOC6	85	28%	29	16%	1.7						
GNS	42	14%	24	13%	1.0						
CHAC1	72	24%	24	13%	1.8						
CLGN	46	15%	5	3%	5.4						
KIF1A	42	14%	3	2%	8.3						
BTG1	205	67%	95	53%	1.3						
SOD3	14	5%	7	4%	1.2						
RMND5B	29	10%	15	8%	1.1						
ZCCHC3	53	17%	10	6%	3.1						
*Total No.	305		180			*Total No.	305		180		

*Indicates number of estimated cancer cells.

We investigated whether malignant cells expressing stem cell markers and those in an EMT transcriptional state coexisted in the E-side and M-side. The analysis showed that the three types of cancer cells (EA, EA^intEMT^ and EA^EMT^) expressed SOX2, suggesting that cancer stem-like cells might become EMT cells (Fig. 5f). In addition, cells expressing other Sph-specific markers, such as CTSV, S100B, and LCP1, were present at a higher rate in the E-side, suggesting that the E-side contained cells with greater tumorigenic activity. Notably, the E-side, but not the M-side, contains active tumorigenic cells and EMT cells. In contrast, T cells infiltrated into the M-side at a higher rate than into the E-side.

### Origin of the squamous cell carcinoma

**Figure 6:**
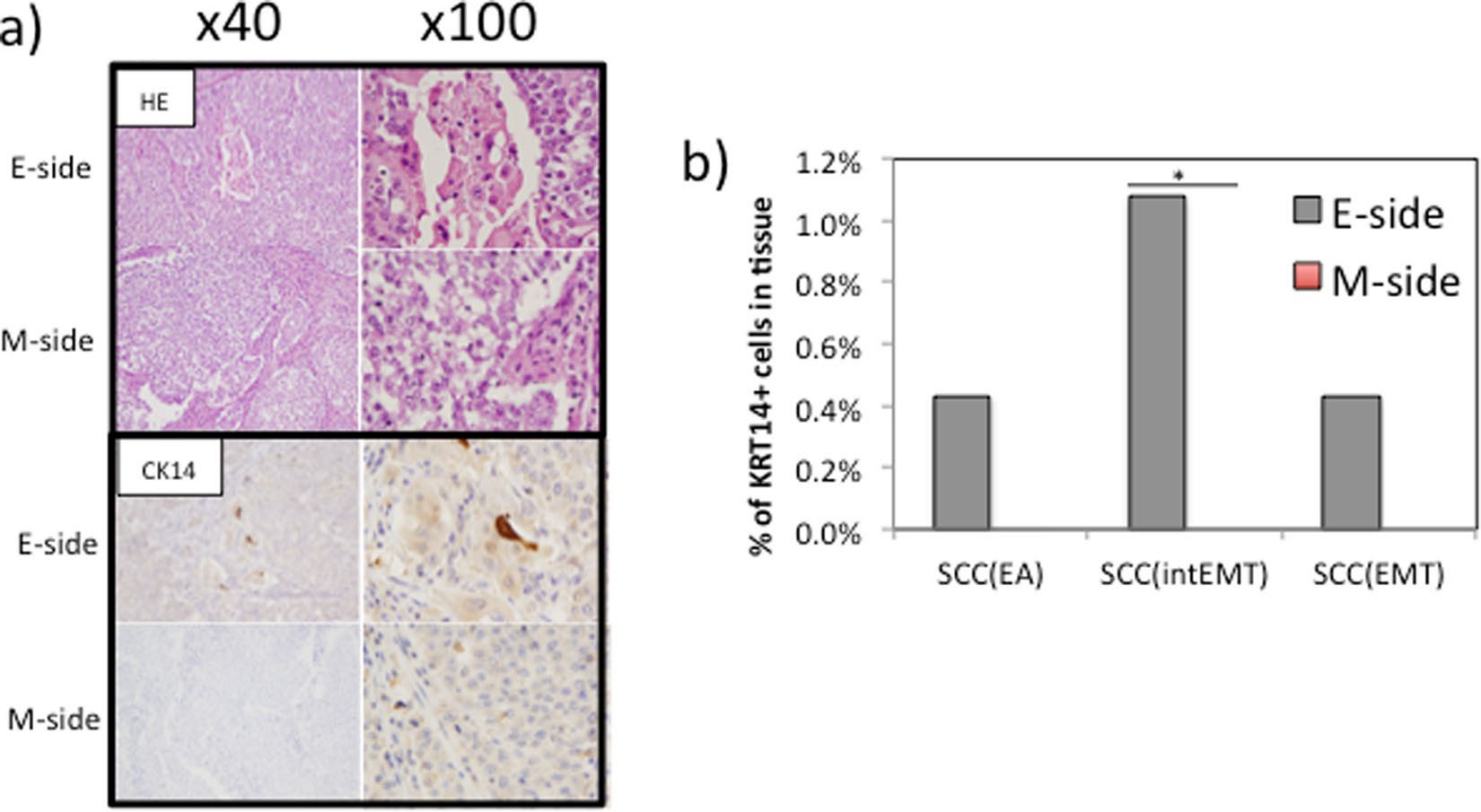
Squamous cell carcinoma from an endometrioid adenocarcinoma shows positive staining for CK14 is present only in the E-side. (**a**) Hematoxylin and eosin staining of the E-side and M-side (upper) and after the application of the CK14 antibody (lower). A higher number of CK14 positive cells are present in the E-side. Original magnification 40x and 100x. (**b**) KRT14 positive cells are only present in the E-side. *p < 0.05 for E-side versus M-side by the Mann–Whitney *U*-test.

The origin of squamous cell carcinoma (SCC) in endometrioid adenocarcinoma with squamous differentiation is not clear. Because KRT14 is a marker for squamous cell carcinoma^23^, we screened the gene expression of KRT14-positive cells. The results indicated that KRT14-positive cells were present in the E-side but not in the M-side (Fig. 6a,b). This finding was confirmed by high molecular weight keratin staining. Single cell gene analysis showed that some SCCs positive for KRT14 expressed the adenocarcinoma marker KRT8 as well as the EMT markers VIM, and ZEB2 (Fig. 6b).

Bass *et al*.^24^ reported that SOX2-driven tumors expressed markers of both squamous differentiation and pluripotency. In the current study, one cell expressing KRT14 was found among SOX2-positive cancer cells in the E-side. This cell was also positive for BIRC5, STMN1, SOX2, KRT14, VIM, CD24, EPCAM1 and ZBTB17. Overall, our data suggested that the gene expression of KRT14 (but not the antibody) is a marker for SCCs in endometrial cancer. Moreover, SCC may be related to EMT and associated with factors such as serum or attachment signals.

## Discussion

Cancer cells display a range of phenotypic states that differ in their functional properties. It has long been postulated that intratumoral heterogeneity contributes to disease progression and has a marked effect on therapeutic efficacy. Previous studies have shown the heterogeneity of cancer cells and stroma cells. Our newly developed single cell transcriptome analysis, which we termed Nx1-seq, can overcome issues related to heterogeneity and provide novel insights into tumor microenvironments. This new approach is simple and can be used to analyze several hundreds to tens of thousands of cells without special equipment. Moreover, these microwells with barcode beads in a Lab-Tek chamber slide can be stored with buffer for several months before use. Therefore, the plate can be carried anywhere and analyses can be performed immediately after cell isolation without the need to prepare plates. In addition, the size of the well can be modified depending on the size of the target cells, larger microwells for cancer cells or smaller microwells for leukocytes and other non-cancerous cells, or with varying degrees of morphology and membrane composition. To determine whether cells are present in a well, empty wells without beads can be determined by light microscopy. Alternatively, by using emulsion (em)PCR, we placed an insert sequence for any binding site such as TCR, or IgG, into the capture beads using specific primers (Supplementary Fig. 5).

In this study, we characterized complex heterogeneous cell samples from human endometrioid adenocarcinoma tissue and analyzed thousands of cells per experiment using Nx1-seq. Human endometrioid adenocarcinoma tissues are comprised of varying ratios of different cell populations of infiltrating immune cells, fibroblasts and cancer cells. Here, we also investigated the differences in cells from the E-side and M-side. Myometrial invasion is an independent prognostic parameter of endometrioid carcinomas and is correlated with the risk of metastasis to the lymph nodes. In addition, cells expressing malignancy-related genes present at greater numbers in the myoinvasive front. However, in this study, cancer cells in the E-side were highly malignant compared with those in the M-side. Many cells on the E-side were positive for spheroid-specific markers, such as *SOX2*, *CTSV* and *GNG2*, which are related to tumorigenesis. However, EMT is also regulated by multiple epigenetic mechanisms that can repress the expression of epithelial markers and convert epithelial cells into aggressive, invasive tumor cells with stem cell properties. In addition to the increased frequency of cells with a cancer stem cell marker in the E-side, the population of EA^intEMT^ and EA^EMT^ cells in the E-side was higher than in the M-side. Moreover, the population of infiltrating T cells in the tumor, which is a marker for prognosis, was smaller in the E-side compared with the M-side. These data obtained by Nx1-seq, demonstrated that cells with high malignant potential (HMP) were present in the site of the same cancer tissue.

Examination of gene expression in sphere cells identified *TCEAL4* ^25^, *BAG1* ^26^, *VAT1* ^27^ and *GPI* ^28^ genes, which were significantly more highly expressed in EA^EMT^ than EA cells. These genes were previous reported as cancer markers in gynecological tumors. Therefore, they may be useful as HMP markers of endometrial adenocarcinoma.

Huvila *et al*. examined the biological differences between low-grade and high-grade endometrioid endometrial adenocarcinomas^29^ and Mhawech-Fauceglia *et al*. investigated the differentially expressed genes between late and early stage and good prognosis vs. poor prognosis endometrioid adenocarcinoma^30^. When we compared our data with these data sets, only *GPI* was consistently observed in both studies. Why so few genes were detected might be related to the difference in individuals and/or cancer stage or these specific genes might only be expressed in rare cells observed by single cell analysis.

**Figure 7:**
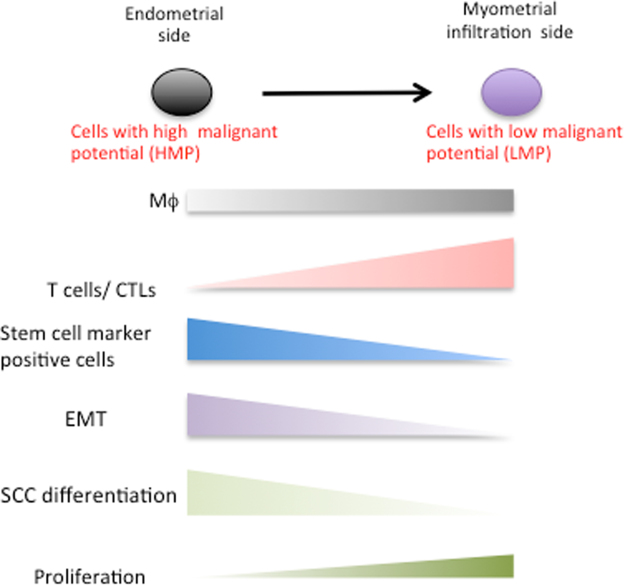
Diversity status of the endometrial adenocarcinoma.

We hypothesized that sites with two distinct characteristics were present in this carcinoma tissue: cancer cells at an HMP site, and a second site where the HMP of the cancer cells had decreased (Fig. 7). Cancer cells in the E-side had reduced proliferation potency similar to that of stem-like cells. This site may be a niche for cancer stem-like cells, and may be more prone to EMT. However, immunocytic invasion occurs at an extremely low frequency in this site. At the M-side, the expression of chemokines increases and immunocytic invasion occurs. Therefore, to develop more effective cancer therapies, our hypothesis suggests that the diversity of cancer cells in each distinct site of tumor tissues must be considered.

In conclusion, Nx1-seq analysis is a powerful approach for characterizing and understanding cellular diversity under physiological or pathological conditions. We showed that a cancer tissue in endometrioid adenocarcinoma with squamous differentiation was heterogeneous with a wide range of immune cells.

## Methods

### Clinical specimens

The endo`metrioid adenocarcinoma samples were obtained with informed consent from patients who had undergone radical resection at the Sapporo Medical University Hospital (Sapporo, Japan), and tissue acquisition procedures were approved by the ethics committee of Sapporo Medical University. Blood samples were obtained with informed consent from a healthy volunteer and collection was approved by the ethics committee of Sapporo Medical University. All methods were carried out in according with the relevant guidelines of the ethics committee of Sapporo Medical University and Kanazawa University.

### Tumor sample

Endometrioid adenocarcinomas with squamous differentiation (grade III at diagnosis) were obtained from a patient and minced. The cell mixture was digested with collagenase and dispase, and cultured in PBS(-). A single cell suspension was passed through a 30-μm nylon mesh, and live cells were isolated by density gradient centrifugation using Lymphoprep (Axis Shield, Oslo, Norway) at 780 × g for 20 min.

### Development of a method for comprehensive single cell transcriptome analysis

Our method for single cell transcriptome analysis can provides digital gene expression data for hundreds or thousands of single cells. The method is a scalable approach for digital gene expression profiling of thousands of single cells without the use of robotics. The method has three steps: 1) preparation of polystyrene beads conjugated with barcode nucleotides and of oligo-dTs by means of emulsion PCR (Supplementary Fig. 1a); 2) placing a barcoded bead into a well and mixing a single cell with a barcoded bead; 3) adding a population of heterogeneous cells and beads into 20-pL wells molded in polydimethylsiloxane (PDMS) slides (Supplementary Fig. 1b). Each slide can contain 1.6 × 10^5^ wells (2 × 2 mm). Poly(dT) barcoded beads with a diameter of 20 μm were first added to the microwell slide to achieve 1 bead/well. Approximately 10,000 cells were allowed to settle into the wells of a PDMS slide by gravity. The slides were covered with dialysis membrane and then incubated with a cell lysis solution containing 1% lithium dodecyl sulfate. After lysis, cellular mRNA was bound to the poly(dT) barcoded beads, which were then collected and used for reverse transcription.

### Preparation of barcode-dT capture beads

The protocol for preparing barcoded beads was modified slightly from that described in the instruction manual for the GS Junior Titanium emPCR Kit (Lib-L) kit (Roche) as shown in Supplementary Fig. 6. Briefly, the synthesized oligonucleotide (5′-CCATCTCATCCCTGCGTGTCTCCGACTCAGGCAGTGAAAAAAAAAAAAAAAAAAAAAAAAANNNNNNNNNNNNACATAGGCCGTCTTCAGCCGCTGAGACTGCCAAGGCACACAGGGGATAGG-3′, 1 × 10^5^ molecules) was hybridized to polystyrene DNA capture beads (1 × 10^7^ beads) bound to specific oligonucleotide (5′-CCTATCCCCTGTGTGCCTTGGCA-3) included in the kit and clonally amplified by emulsion PCR with primer-1 (5′-CCTATCCCCTGTGTGCCTTGGCA-3′) and primer-2 (5′ biotin–18-atom hexa-ethyleneglycol spacer -CCATCTCATCCCTGCGTGTC-3′) (IDT Technologies). After breaking the emulsion, an enrichment process was utilized to selectively capture beads with an oligo-dT, while rejecting null beads. After the beads were purified, they were treated with *ExoI*(NEB) for 30 min. The *ExoI* was inactivated at 80 °C, and the beads were incubated with *BtsI*(NEB) for 2 hours. Next, the enriched double-stranded DNAs on the capture beads were cut with *BtsI* for 2 hours at 55 °C. After digestion, double strand DNA on the beads was rendered single stranded by removal of the secondary strand through incubation in 0.125 M NaOH. The supernatant was carefully removed and discarded. The residual solution was then diluted by the addition of 1 mL annealing buffer (20 mM tris-acetate, pH 7.6, 5 mM magnesium acetate), and the suspension was vortexed at a medium speed for 2 seconds; the beads were then pelleted and the supernatant removed. The beads were stored at −20 °C until use.

The barcodes on the beads were produced using emulsion PCR using randomly synthesized barcode oligo DNAs. Because multiple beads can enter the water-in-oil (w/o) emulsion droplet that acts as the reaction compartment, we examined the diversity of the barcodes. The DNA of several barcoding beads was sequenced using a GS Junior Titanium sequencer to confirm whether a single barcode was multiplied after making the barcoded beads. The data indicated that overlapping barcodes in single wells accounted for less than 2% of the 10,000 beads (Supplementary Fig. 1c). Therefore, the barcoded beads were suitable for single cell analyses.

### Preparation of microwell slides

A grid of micropillars (25-μm diameter, 40-μm height) was photolithographically patterned onto a silica wafer using SU-8 photoresist. The silica wafer was then used as a mold to print the PDMS slides (SILPOT 184, Dow Corning Toray Co., Ltd., Japan); these slides have the dimensions of a standard microscope slide and each contains ~160,000 wells in a 20 × 20 mm section. The molded PDMS slides were placed in an oxygen plasma chamber (YSR, SAKIGAKE Semiconductor Co., Ltd) for 5 min to generate a hydrophilic surface. The slides were then set into Lab-Tek® Chamber Slide (2 well style, Thermo Fisher Scientific) The slides were then blocked in 1% bovine serum albumin (BSA) for 30 min and washed with deionized water followed by PBS to prepare them for cell seeding.

Barcoded beads (~600,000) in 1.0 mL PBS were allowed to settle into the wells of a PDMS slide by gravity over the course of 30 min with gentle agitation to distribute the suspension over the slide surface. To ensure one bead per well, a BSA-blocked dialysis membrane (12,000–14,000 MWCO regenerated cellulose, Spectra/Por 2 membrane discs, φ47 mm, Spectrum), that had been rinsed with PBS, was placed on the slide surface to seal the microwells. Excess beads in wells were removed by squeezing the surface of the slide with a small stick. Then, the presence of one bead per well was determined using a microscope.

### Synthesis of cDNA of a single cell

A suspension of 10,000 cells was placed on each PDMS microwell slide (~160,000 wells) and allowed to settle into the wells by gravity over the course of 15 min with gentle agitation. The cell seeding process was calculated to be 90% efficient by measuring the cell concentration in seeding buffers for both pre- and post-cell seeding. The distribution of cells as single or multiple cells per well was calculated using Poisson statistics. The cell:well ratio of 1:20 used in this experiment corresponds to 95% of cells being deposited at one cell/well. The cells were allowed to settle into wells, then a BSA-blocked dialysis membrane rinsed with PBS was placed on the slide surface, sealing the microwells and trapping the cells and beads inside. Excess PBS was removed from the slide and membrane surfaces using a 200 μL pipette. Cell lysis solution (200 μL of 500 mM LiCl in 100 mM TRIS buffer (pH 7.5) with 1% lithium dodecyl sulfate, 10 mM EDTA and 5 mM DTT) was applied to the dialysis membranes for 12 min at room temperature. Time-lapse microscopy revealed that all cells were fully lysed within 3 min. Then, the dialysis membrane was removed with forceps and discarded. Working quickly, the PDMS slide was inverted in a Petri dish containing 2 mL of cold lysis solution and a magnet was applied beneath the Petri dish to force the beads out of the microwells. Subsequently, 1 mL aliquots of the lysis solution containing the resuspended beads were placed into 1.5 mL tubes and the beads were washed at room temperature for 15 seconds at 10,000 rpm and washed once without resuspension using 1 mL per tube of wash buffer A (100 mM Tris, pH 7.5, 500 mM LiCl, 1 mM EDTA, 4 °C). Beads were resuspended in the wash solution using 100 μL per tube of buffer B (20 mM Tris, pH 7.5, 50 mM KCl, 3 mM MgCl), and then pelleted again.

### Generation of NGS library

First-strand cDNA generation was carried out by adding 5 × First Strand Buffer (250 mM Tris-HCl, pH 8.3, 375 mM KCl and 30 mM MgCl_2_), dithiothreitol (100 mM), dNTP mix (10 mM), RNase inhibitor, oligos (template switching oligo1, 5′-CTCGGCATTCCTGCTGAACCGCTCTTCCGATCTrGrG + G; G: 1 LNA-modified guanosine) and SuperScript II Reverse Transcriptase in a total volume of 30 μL. After first-strand synthesis, cDNA was amplified in a volume of 50 μL by PCR using 16 pmol primer A (5′-/5BioTEG/GCGGCTGAAGACGGCCTATGT-3’) and primer B (5′-CTCGGCATTCCTGCTGAACCGCTCTTCCGATCT-3’). The cDNA was produced using 18 cycles of 98 °C for 1 min, 60 °C for 1 min, 68 °C for 4 min, and an additional cycle at 72 °C for 10 min. The PCR product was sonicated and the DNA was then run on a 1% agarose gel. A 300–500-bp band was purified. This DNA was mixed with adaptor 1 (5′-GTGACTGGAGTTCAGACGTGTGCTCTTCCGATCT-3’) and adaptor 2 (5′-/5Phos/AGATCGGAAGAGCACACGTCTGAACTCCAGTCAC/3AmMC7/-3′) with T4 DNA ligase, and the mixture was incubated at 16 °C for 12 h. After purification, the purified DNA was amplified by PCR using the primers 5′-AATGATACGGCGACCACCGAGATCTACACTCTTTCCCTACACGACGCTCTTCCGATCTGCGGCTGAAGACGGCCTATGT-3′and 5′ AATGATACGGCGACCACCGAGATCTACACTCTTTCCCTACACGACGCTCTTCCGATCT-3′. The 50-μL mixture for PCR contained 200 nM of each primer, 200 nM, dNTP, PS buffer, and 1.25 units PrimeSTAR HS DNA polymerase (TaKaRa) and was run at 98 °C for 30 s, followed by 12 cycles at 98 °C for 15 s, 62 °C for 15 s, 72 °C for 1 min, and then 72 °C for 10 min. The PCR product was run on a 1% agarose gel. The purified libraries were sequenced with the HiSeq. 2000 system and Miseq, following the manufacturer’s protocol.

Mapping and annotation was performed using bowtie2 software and Perl custom scripts. Read 1 contained the barcode sequence (12 N) and primer sequence following it, otherwise the read 2 sequence was from each cell mRNA. The barcode sequences were extracted from the read 1 fastq file. The read 2 sequences were aligned against RefSeq mRNA (ftp://ftp.ncbi.nih.gov/refseq/H_sapiens/mRNA_Prot/) as a reference sequence using bowtie 2 software. After mapping, the barcode was linked to its paired read 2 alignment data, and genes were counted for each barcode. One thousand cells from the E-side and M-side were analyzed and yielded more than 20,000 reads and more than 400 genes per cell (range about 1,039-8126 genes per cell in E-side and about 420-8,814 genes per cell in M-side). The total number of tags from each cells in E-side and M-side was normalized to 300,000.

### Immunohistochemical staining

Immunohistochemical staining was performed using formalin-fixed, paraffin-embedded sections. Sections (4-5 µm thick) were deparaffinized in xylene and rehydrated in graded alcohols and antigen retrieval was performed using target retrieval solution (pH 9.0) (DAKO, Tokyo, Japan). Endogenous peroxidase activity was blocked by 3% hydrogen peroxide in ethanol for 10 min. The sections were incubated with primary antibody (Supplementary Table 6) for 1 hour followed by incubation with biotinylated anti-mouse IgG or anti-rabbit IgG (Nichirei, Tokyo, Japan) for 30 min. Subsequently, the sections were stained with streptavidin-biotin complex (Nichirei), followed by incubation with 3,3′-diaminobenzidine as the chromogen and counterstaining with hematoxylin.

### Spheroid formation assay

Sphere cultures were performed as described previously^31^. Cells were plated at 1000 cells per well in six-well ultra-low attachment plates (Corning Inc., Corning, NY) and cultured in a DMEM/F12 medium with 10 ng/mL hEGF, 10 ng/mL hbFGF, and 2% B-27 (Life Technologies Corp.) at 37 °C in 5% CO_2_. On day 7, the number of colonies was counted under an inverted contrast microscope.

### Xenograft

Spheroid attached cells were collected and resuspended at 1 × 10^2^ to 1 × 10^5^ cells per 50 µL of PBS, followed by the addition of 50 µL of Matrigel (BD Biosciences). This cell-Matrigel suspension was subcutaneously injected into the backs of 4–6-week-old NSG mice (NOD.Cg-*Prkdc* ^*scid*^ *Il2rg* ^*tm1Wjl*^/SzJ, Charles River Laboratory, Yokohama, Japan) under anesthesia. Tumor growth was monitored weekly for 12 weeks. Then, the xenografted tumors were resected and analyzed for the tumorigenicity of sphere cells at the subcutaneous injection site.

### Clustering analysis and cell classification

t-Distributed Stochastic Neighbor Embedding (tSNE) analysis^32^ and visualization was performed using R. Cancer and other cells were also defined as described in Supplementary Table 2.

### Statistical analysis

The Pearson correlation coefficient was calculated to determine the correlation between a cell versus bulk library, experiment 1 versus experiment 2, and each cell marker. Pair-wise multiple group comparisons were analyzed by the Mann-Whitney U-test with FDR correction by Benjamini/Hochberg method.

### Accession Numbers

The Nx1-seq data have been deposited in the DNA Databank of Japan (DDBJ) with the accession number DRA005192.

## Electronic supplementary material

Supplementary Figure

Supplementary Table 1

Supplementary Table 2

Supplementary Table 3

Supplementary Table 4

Supplementary Table 5

Supplementary Table 6
