# Supplementary material for: Comprehensive single-cell transcriptome analysis reveals heterogeneity in endometrioid adenocarcinoma tissues
Source: Sci Rep. 2017 Oct 27;7:14225. doi: 10.1038/s41598-017-14676-3 (PMC5660171; doi:10.1038/s41598-017-14676-3)

**Supplementary Information**

**Comprehensive single-cell transcriptome analysis reveals heterogeneity in endometrioid adenocarcinoma tissues**

Shinichi Hashimoto^1),2)*^, Yuta Tabuchi^3)^, Hideaki Yurino^1),2)^, Yoshihiko Hirohashi^2),3)^, Shungo Deshimaru^4)^, Takuya Asano^3)^, Tasuku Mariya^3)^, Kenshiro Oshima^5)^, Yuzuru Takamura^6)^, Yoshiaki Ukita^7)^, Akio Ametani^4)^, Naoto Kondo^8)^, Norikazu Monma^9)^, Tadayuki Takeda^8)^, Sadahiko Misu ^9)^, Toshitugu Okayama^9)^, Kazuho Ikeo^9)^, Tsuyoshi Saito^10)^, Shuich Kaneko^11)^, Yutaka Suzuki^5)^, Masahira Hattori^5),12)^, Kouji Matsushima^4)^, Toshihiko Torigoe^2),3)^

*Corresponding author: Shinichi Hashimoto

Department of Integrative Medicine for Longevity, Graduate School of Medical Sciences, Kanazawa University, 13-1 Takaramachi, Kanazawa, Ishikawa 920-8641, Japan

**Supplementary Figures and legends**


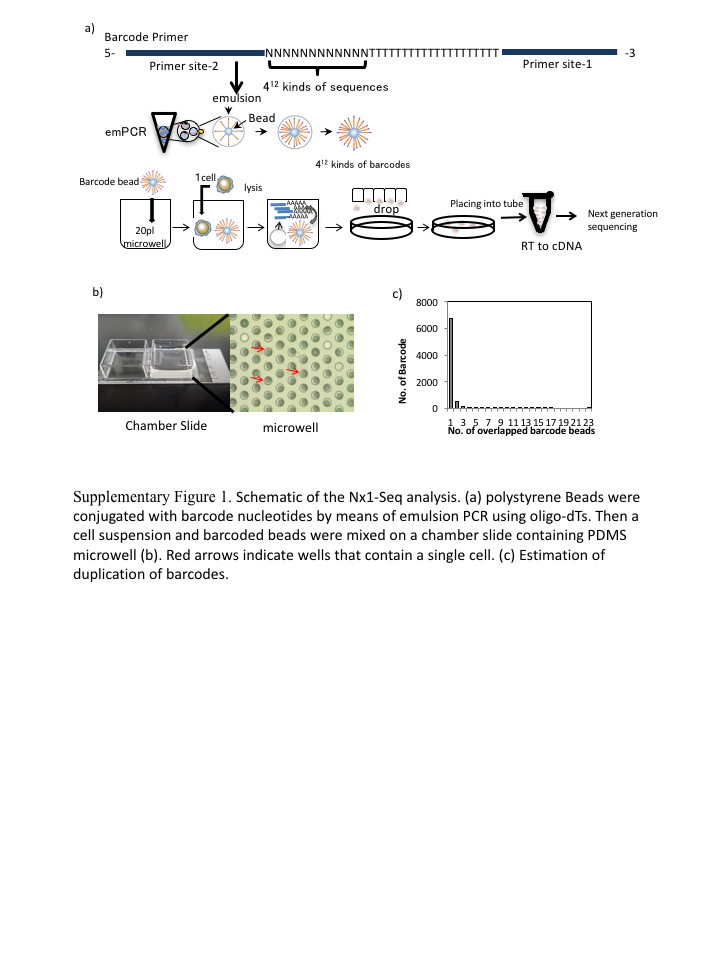


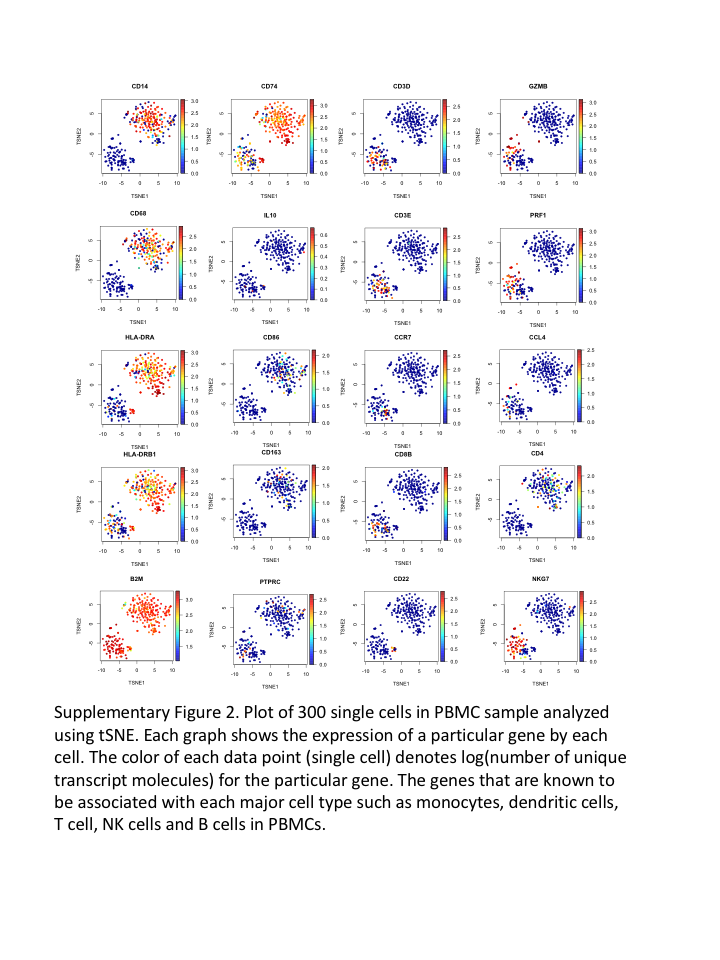


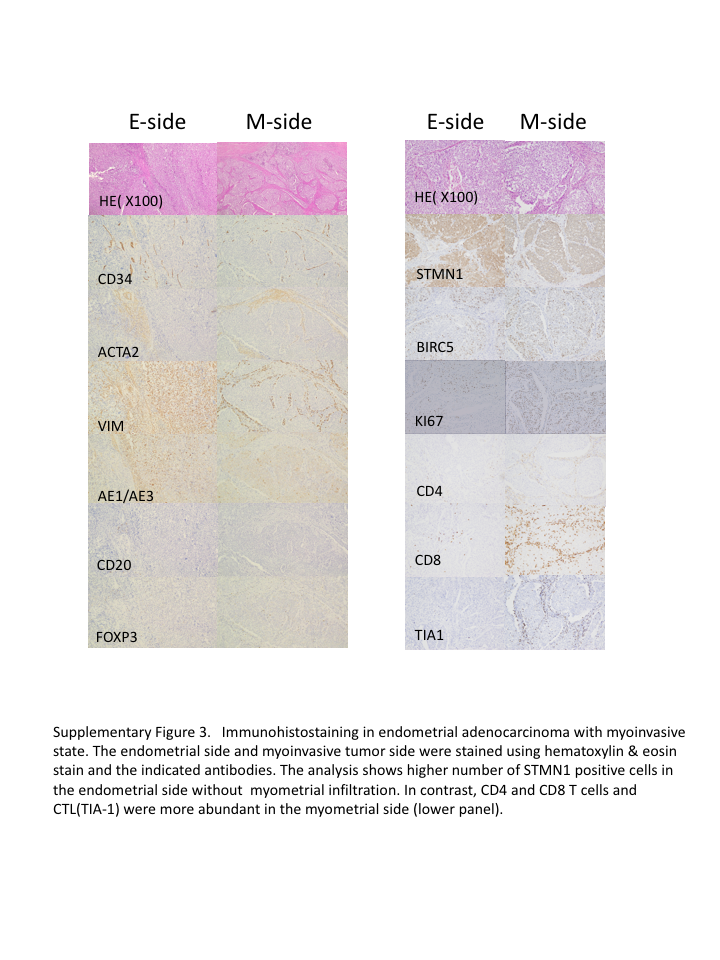

Supplement: Supplementary file 1 — Supplementary Figure [file 41598_2017_14676_MOESM1_ESM.doc]
